# Modeling autonomous shifts between focus state and mind-wandering using a predictive-coding-inspired variational recurrent neural network

**DOI:** 10.3389/fncom.2025.1578135

**Published:** 2025-07-02

**Authors:** Henrique Oyama, Takazumi Matsumoto, Jun Tani

**Affiliations:** Cognitive Neurorobotics Research Unit, Okinawa Institute of Science and Technology Graduate University, Okinawa, Onna, Japan

**Keywords:** mind-wandering, predictive coding, free energy principle, variational RNN, brain-inspired modeling

## Abstract

Mind-wandering reflects a dynamic interplay between focused attention and off-task mental states. Despite its relevance in understanding fundamental cognitive processes, such as attention regulation, decision-making, and creativity, previous models have not yet provided an account of the neural mechanisms for autonomous shifts between focus state (FS) and mind-wandering (MW). To address this, we conduct model simulation experiments employing predictive coding as a theoretical framework of perception to investigate possible neural mechanisms underlying these autonomous shifts between the two states. In particular, we modeled perception processes of continuous sensory sequences using our previously proposed variational RNN model under free energy minimization. The current study extends this model by introducing an online adaptation mechanism of a meta-level parameter, referred to as the meta-prior **w**, which regulates the complexity term in the free energy minimization. Our simulation experiments demonstrated that autonomous shifts between FS and MW take place when **w** switches between low and high values responding to a decrease and increase of the average reconstruction error over a past time window. Particularly, high **w** prioritized top-down predictions while low **w** emphasized bottom-up sensations. In this work, we speculate that self-awareness of MW may occur when the error signal accumulated over time exceeds a certain threshold. Finally, this paper explores how our experiment results align with existing studies and highlights their potential for future research.

## 1 Introduction

During mindfulness practice, such as focusing on sensations like breathing, our attention sometimes spontaneously deviates to mental imagery or thoughts about the past and future, a phenomenon known as mind-wandering (Smallwood and Schooler, 2015; Christoff et al., 2016; Seli et al., 2016). This shift from a focused state to mind-wandering can occur not only during meditation but also in everyday activities, such as driving, listening to music, or tasting food. Mind-wandering tends to occur more frequently during tasks that are either too easy or too difficult. When tasks are less demanding, such as simply attending to breathing, instances of mind-wandering increase. Conversely, during more challenging tasks, such as reading complex material, our minds are more prone to wander because maintaining focus becomes difficult over long periods (Seli et al., 2018; Peral-Fuster et al., 2023).

An interesting aspect is that the transition from the focused state (FS) to the mind-wandering state (MW) often happens without conscious awareness, whereas the shift from MW back to FS involves recognizing the mind-wandering episode consciously (Smallwood and Andrews-Hanna, 2013). Various studies have investigated the psychological and systematic mechanisms underlying these shifts. For example, Henŕıquez et al. (2016) argued that the transition from FS to MW is gradual, as evidenced by increasing response times during focused tasks. In contrast, Vago and Zeidan (2016) suggested that the shift is abrupt, triggered by sudden internal or external stimuli.

Voss et al. (2018) proposed a model where mental states alternate between FS and MW, with MW episodes ending when individuals consciously recognize their mind-wandering and return to the task. This “two-stage model” assumes that the probability of being in FS is higher at the beginning of an episode and decreases over time. However, contrary to this prediction, Zukosky and Wang (2021) found that the probability of FS does not decline within an FS-MW episode in a subject study introducing a probe at a random time during the episode. To address this discrepancy, the authors proposed the “multiple sub-event model,” which hypothesizes that unconscious alternations between FS and MW occur multiple times before an individual becomes aware of being in MW. Their simulation study suggested that as the number of sub-sequences increases, the decline in the probability of FS becomes less pronounced.

Although the studies mentioned above clarified some phenomena in the shift from FS to MW from psychological observation, they have not provided sufficient accounts for the underlying neuronal mechanisms. Recently, some studies (Sandved-Smith et al., 2021; Idei et al., 2024) suggested system level neuroscience models incorporated with the concept of the free energy principle (FEP) (Friston, 2005). Here, FEP is briefly explained for better understanding of the readers. The FEP is a neuroscience theory that has attracted large attention. The FEP posits that humans and animals execute various functions such as learning, perception, and action generation to maximize their chances of survival by minimizing surprises they encounter during interaction with the environment. According to the FEP, these functions are achieved by optimizing generative models for predicting the sensation, whereby a common statistical quantity called free energy is minimized. The FEP supports two frameworks, one is predictive coding and the other is active inference. Predictive coding provides a formalism accounting for how agents perceive sensations. It suggests that the brain predicts sensory observations in the top-down pathway, while at the same time updating posterior beliefs about those sensations in the bottom-up pathway whenever errors arise between predictions and observations (Rao and Ballard, 1999; Friston, 2005; Clark, 2015). By updating posterior beliefs in the direction of minimizing errors, perceptual inference for the observed sensation can be achieved. On the other hand, active inference (AIF) provides a theory for action generation by assuming that the brain is embodied deeply and embedded in the environment, such that acting on it changes future sensory observation. Then, AIF considers that actions should be selected such that the error between the desired and predicted sensations can be minimized (Friston et al., 2010, 2011).

Sandved-Smith et al. (2021) postures the underlying mechanism of the shift from FS to MW using active inference of “mental action” in terms of attention changes. The proposed model assumes a hierarchical probabilistic generative model wherein the hidden meta-awareness states in the higher level account for “how aware am I of where my attention is?,” the hidden mental states in the middle level dealing with focus of attention account for “what am I paying attention to?,” and the sensorimotor hidden states in the lowest level do for “what am I perceiving or trying to do?” according to the authors. The states at each level condition the ones in the next lower level by controlling their precisions or beliefs. Agent's perceptual and attentional states are inferred at each time step by means of active inference in minimizing the expected free energy. The results of simulation experiments show that when the meta-awareness state is manually shifted from high to low, distracted or MW state is developed more frequently. Under this condition, redirection back to FS by consciously being aware of the current MW state tends to take more time because of less precision in the attention toward distracted state.

Idei et al. (2024) investigated mind-wandering mechanism by conducting a model simulation study on allostasis using a hierarchically organized variational recurrent neural network, so-called the PV-RNN (Ahmadi and Tani, 2019). Dynamic behavior of PV-RNN can be characterized by a meta-level parameter, referred to as meta-prior **w**, that regulates the complexity term against the accuracy term in free energy which is minimized in the inference of the posterior probability distribution of the latent variables. It was shown that a high setting of meta-prior **w** enhances the generation of the top-down imagery while a low setting of it enhances the bottom-up sensory perception (Ohata and Tani, 2020; Chame et al., 2020; Wirkuttis et al., 2023). Analogous to this, Idei et al. (2024) showed that a low setting of **w** generates stronger sensory bottom-up which leads to FS wherein less change in movement as well as neural activity are observed. On the other hand, high setting of **w** generates weak attention to sensation and stronger top-down processing which leads to MW wherein more movement as well as neural activity.

The aforementioned FEP-based studies provide valuable insights into macroscopic neural mechanisms, such as redirecting attention to focused states by inferring one's attentional state, or generating mind-wandering by balancing top-down and bottom-up information flows. However, these studies do not provide systematic explanations for how the shifts between FS and MW could be autonomously generated, since the shift from FS to MW in Sandved-Smith et al. (2021) is caused by manual change of the meta-aware state from high to low and the one in Idei et al. (2024) does this by resetting meta-prior **w** from low to high value.

In this regard, the current study speculates that autonomous transition between FS and MW could be generated by introducing an online adaptation mechanism of meta-prior **w** to PV-RNN in which **w** is modulated with response to some macroscopic variables such as an average reconstruction error. In our study, PV-RNN learns to predict a target sequence of continuously changing sensory patterns which is generated by means of predetermined probabilistic transitions among a set of cyclic patterns. In the test phase after the training, given one of the pre-trained cyclic patterns as the target inputs, the PV-RNN predicts encountering sensory inputs by simultaneously inferring the approximated posterior of the latent state at each time step by minimizing the free energy while adapting **w**. Analogous to studies (Ohata and Tani, 2020; Chame et al., 2020; Wirkuttis et al., 2023; Idei et al., 2024), when **w** modulates to a lower value by reflecting the surge of the average reconstruction error, the inference process may improve by placing greater emphasis on bottom-up sensations. This situation may correspond to FS. On the other hand, when **w** modulates to a higher value by responding to the decline of the average reconstruction error, the PV-RNN may generate top-down imagery by following the learned probabilistic transitions of the patterns while ignoring the target sensory inputs. This may correspond to MW. Our simulation study with PV-RNN under various parameter settings will evaluate this hypothesis. The following section introduces the proposed model, followed by a detailed description of the simulation experiment setup, the presentation of the results, and a discussion that includes proposals for extensions to future research work.

## 2 Materials and methods

### 2.1 Overview

This study investigates autonomous shifts between the focused state (FS) and mind-wandering (MW) during a perception task using sequential sensory input patterns. The predictive coding framework is employed to model this perception process. Predictive coding assumes a generative model that predicts sensory sequences by learning both the latent state transition function and the likelihood mapping from latent states to sensory observations. Additionally, this generative model infers the current latent state through continuous sensory sequence observations.

Both learning and inference processes are achieved by minimizing reconstruction error, or more specifically, free energy. We hypothesize that FS is enhanced by strengthening bottom-up inference, while MW becomes more likely by emphasizing top-down sensory pattern generation. It is also hypothesized that shifts between FS and MW take place autonomously by incorporating an online adaptation of meta-level states in response to particular system variables. To test this, we propose an extended version of a variational recurrent neural network model, referred to as the Predictive Coding Inspired Variational RNN (PV-RNN) (Ahmadi and Tani, 2019). Details of the original PV-RNN and its extensions are provided in the following sections.

### 2.2 Predictive coding inspired variational RNN model

The PV-RNN is based on the free energy principle (Friston, 2005), where learning and inference are achieved by minimizing free energy (Equation 1) in accordance with Bayes' theorem:


(1) $$ℱ=\underset{\text{complexity}}{\underset{︸}{D_{\text{KL}}[q_{\phi }(z|X)\|p_{\theta }(z)]}}-\underset{\text{accuracy}}{\underset{︸}{E_{q_{\phi }(z|X)}[\log p_{\theta }(X|z)]}}$$

Here, *p*_θ_(**X**) is the marginal likelihood of the sensory observation **X**, given the generative model *p*_θ_ parameterized by θ. The latent variables **z** and inference model *q*_ϕ_, parameterized by ϕ, allow for posterior inference through minimization of free energy. Free energy consists of two terms: the complexity term (a measure of divergence between prior and posterior distributions) and the accuracy term (log-likelihood of sensory observations) (Friston, 2010). PV-RNN serves as both a generative model and an inference model. The generative model predicts future sensory inputs via top-down processes, while the inference model estimates the approximate posterior from observed sensory sequences through free energy minimization as bottom-up processes.

The following subsections describe the PV-RNN implementation and the use of the meta-prior **w**.

#### 2.2.1 Model implementation

The free energy ℱ for PV-RNN predicting a time series of *T* steps is given by:


(2) $$\begin{array}{l} ℱ=\underset{\text{complexity}}{\underset{︸}{w\sum_{t=1}^{T}E_{q_{\phi }(z_{1:t-1}|d_{t-1},X_{t-1:T})}[D_{\text{KL}}[q_{\phi }(z_{t}|d_{t-1},X_{t:T})\|p_{\theta }(z_{t}|d_{t-1})]]}}\\ \;-\underset{\text{accuracy}}{\underset{︸}{\sum_{t=1}^{T}E_{q_{\phi }(z_{1:t-1}|d_{t-1},X_{t:T})}[\log p_{\theta }(X_{t}|d_{t})]}} \end{array}$$

**Figure 1:**
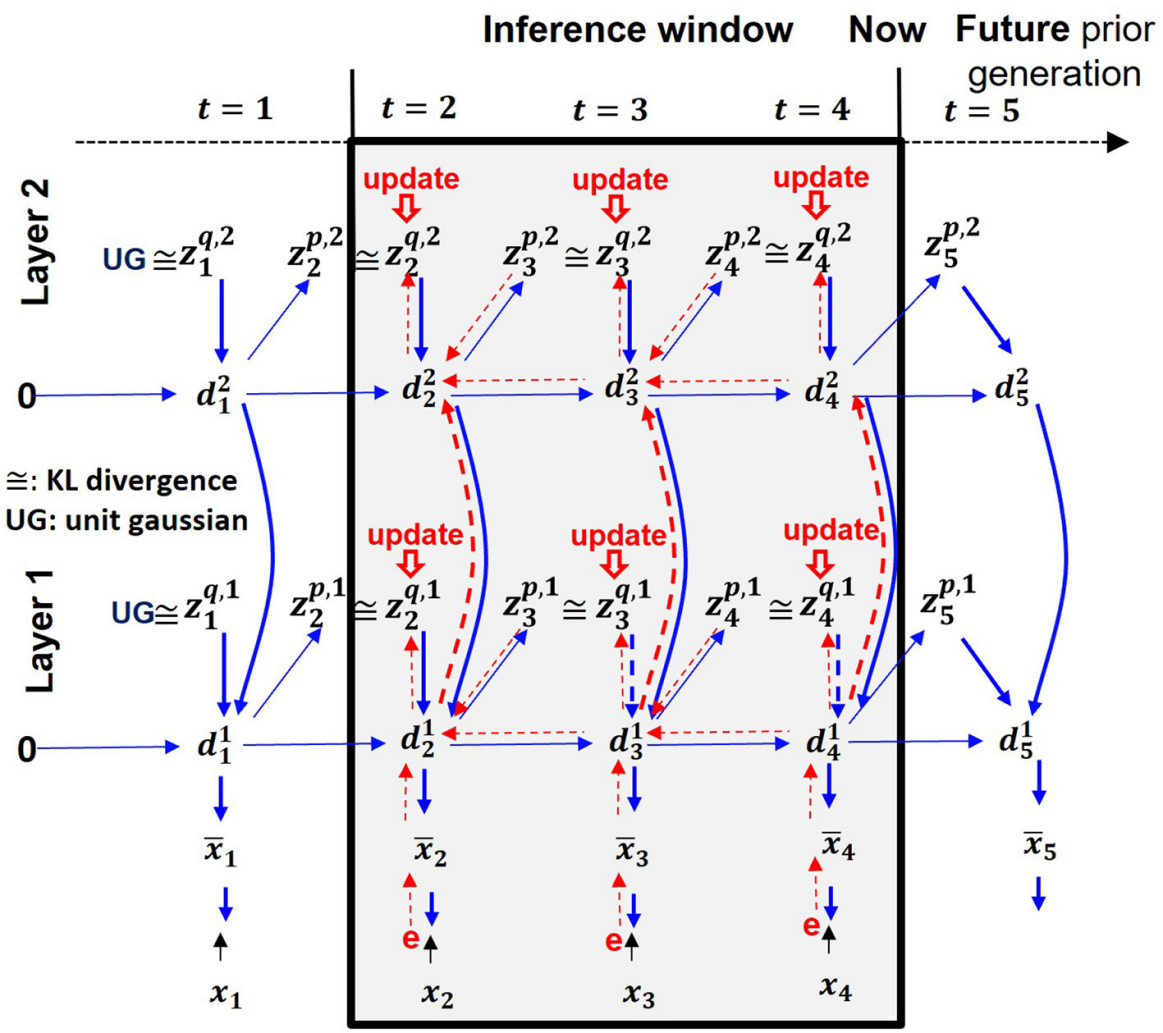
A hierarchical two-layer PV-RNN architecture. Solid blue lines represent the generative process, while dotted red lines indicate the inference process. The shaded area shows an inference window of length 3.

PV-RNN introduces two types of latent variables: probabilistic latent variables (**z**) governed by Gaussian distributions, and deterministic latent variables (**d**). Their relationships are shown in Figure 1. In Equation 2, a meta-level parameter, named meta-prior **w**, is introduced to balance the complexity and accuracy terms during this process. This regulation is particularly important when the limited amount of training data prevents reliable estimation of latent variable distributions. Also, dynamic behavior of PV-RNN is largely affected by the setting of the meta-prior. It was shown that high setting of meta-prior **w** enhances generation of the top-down imagery while low setting of it enhances the bottom-up sensory perception (Ohata and Tani, 2020; Chame et al., 2020; Wirkuttis et al., 2023; Idei et al., 2024).

Next, the forward computation of each variable used in PVRNN is described. At each time step *t*, the internal states of the *l*-th layer (${\text{h}}_{t}^{l}$) are recursively computed:


(3) $$\begin{array}{l} h_{t}^{l}=\left(1-\frac{1}{\tau^{l}}\right)h_{t-1}^{l}\\ \;+\frac{1}{\tau^{l}}\left(W_{dd}^{ll}d_{t-1}^{l}+W_{zd}^{ll}z_{t}^{l}+W_{dd}^{ll+1}d_{t-1}^{l+1}+W_{dd}^{ll-1}d_{t-1}^{l-1}+b_{h}^{l}\right)\\ d_{t}^{l}=\tanh(h_{t}^{l}) \end{array}$$

The PV-RNN structure supports hierarchical information processing using time constants τ^*l*^, enabling the differentiation of temporal dynamics across layers (Yamashita and Tani, 2008; Schillaci et al., 2020).

The generative model computes prior distributions (${\text{z}}_{t}^{p}$) as Gaussian variables parameterized by mean ($\mu_{t}^{p}$) and standard deviation ($\sigma_{t}^{p}$):


(4) $$\begin{array}{l} \mu_{t}^{p}=\tanh(W_{d\mu }^{ll}d_{t-1}+b_{\mu }^{p})\\ \sigma_{t}^{p}=\exp(W_{d\sigma }^{ll}d_{t-1}+b_{\sigma }^{p})\\ z_{t}^{p}=\mu_{t}^{p}+\sigma_{t}^{p}*\epsilon_{t}\text{ with }\epsilon_{t}\sim N(0,I) \end{array}$$

${\text{b}}_{\mu }^{p}$ and ${\text{b}}_{\sigma }^{p}$ are bias terms for $\mu_{t}^{p}$ and $\sigma_{t}^{p}$, respectively. ϵ represents a noise sampled from a standard normal distribution for usage of the reparameterization trick (Kingma and Welling, 2014). Analogous to the computation of the prior distribution, the inference model *q*_ϕ_ approximates the posterior $z_{t}^{q}$ as a Gaussian distribution with mean $\mu_{t}^{q}$ and standard deviation $\sigma_{t}^{q}$.


(5) $$\begin{array}{l} \mu_{t}^{q}=\tanh(W_{d\mu }^{ll}d_{t-1}+A_{t}^{\mu }+b_{\mu }^{q})\\ \sigma_{t}^{q}=\exp(W_{d\sigma }^{ll}d_{t-1}+A_{t}^{\sigma }+b_{\sigma }^{q})\\ z_{t}^{q}=\mu_{t}^{q}+\sigma_{t}^{q}*\epsilon_{t}\text{ with }\epsilon_{t}\sim N(0,I) \end{array}$$

where ${\text{b}}_{\mu }^{q}$ and ${\text{b}}_{\sigma }^{q}$ are bias terms for computing $\mu_{t}^{q}$ and $\sigma_{t}^{q}$, respectively. ${\text{A}}_{t}^{\mu }$ and ${\text{A}}_{t}^{\sigma }$ are adaptive variables to be optimized for inferring the posterior distribution which is parameterized by $\mu_{t}^{q}$ and $\sigma_{t}^{q}$.

Intuitively, the random variable **z**^*p*^ can be regarded as a time-dependent prior/top-down expectation about the encountering sensation. The adaptive vector **A** (i.e., **z**^*q*^) can be regarded as the approximate posterior distribution that may or may not be close to the prior distribution, depending on the setting of meta-prior. **z**^*p*^ and **z**^*q*^ are used by the generative and inference model, respectively to compute the latent variable **d**.

#### 2.2.2 Learning and inference

The free energy ℱ of PV-RNN can be computed as follows by adapting the original (Equation 2). Given a PV-RNN with *L* layers, predicting a *T* time series sensory inputs, ℱ can be written as


(6) $$\begin{array}{l} ℱ=\sum_{t=1}^{T}[\sum_{l=1}^{L}w^{l}D_{\text{KL}}[q_{\phi }(z_{t}^{l}|d_{t-1}^{l},X_{t:T})\|p_{\theta }(z_{t}^{l}|d_{t-1}^{l})]]\\ \;-\sum_{t=1}^{T}\|X_{t}-{\bar{X}}_{t}{\|}_{2}^{2} \end{array}$$

where **w**^*l*^ is meta-prior specific to *l*-th layer, and $\bar{\text{X}}$ denotes the prediction output of the PV-RNN. In Equation 6, we approximate the expectation with respect to the approximate posterior by iterative sampling. Also, the accuracy term is replaced by the squared error, which can be regarded a special case of computation of log-likelihood wherein each dimension of **X** and $\bar{\text{X}}$ is independent and follows a Gaussian distribution with standard deviation 1. Since the Kullback-Leibler (KL) divergence between two one-dimensional Gaussian distributions takes a simple expression, Equation 6 is reduced to


(7) $$ℱ=\sum_{t=1}^{T}[\sum_{l=1}^{L}w^{l}\sum_{r=1}^{R_{z}^{l}}\delta (l,r,t)]-\sum_{t=1}^{T}\|X_{t}-{\bar{X}}_{t}{\|}_{2}^{2}$$

where


(8) $$\delta (l,r,t)=\log\frac{\sigma_{t}^{p,l,r}}{\sigma_{t}^{q,l,r}}+\frac{{\left(\mu_{t}^{q,l,r}-\mu_{t}^{p,l,r}\right)}^{2}+{\left(\sigma_{t}^{q,l,r}\right)}^{2}}{2{\left(\sigma_{t}^{p,l,r}\right)}^{2}}-\frac{1}{2}$$

$\mu_{t}^{p,l,r}$ represents *r*th element of $\mu_{t}^{l}$ of the prior, and the same notation is applied to $\mu_{t}^{q,l,r}$, $\sigma_{t}^{p,l,r}$, and $\sigma_{t}^{q,l,r}$. $R_{z}^{l}$ denotes the dimension of ${\text{z}}_{t}^{l}$. Given that the complexity term is summed over all the dimension of **z**, which is arbitrary to the network design, and the accuracy term is to all the data dimension, which varies among data, the free energy is normalized with respect to the dimension of **z** and the data dimension. Therefore, introducing such normalization, the free energy of PV-RNN in the study is computed by


(9) $$ℱ=\underset{\text{complexity}}{\underset{︸}{\sum_{t=1}^{T}[\sum_{l}^{L}\frac{w^{l}}{R_{z}^{l}}\delta (l,r,t)]}}-\underset{\text{accuracy}}{\underset{︸}{\frac{1}{R_{X}}[\sum_{t=1}^{T}\|X_{t}-{\bar{X}}_{t}{\|}_{2}^{2}]}}$$

where *R*_*X*_ is the data dimension, $R_{z}^{l}$ is the number of **z** variables in each layer, and ${\text{w}}^{l}=R_{z}^{l}{\text{w}}^{l}$. These normalization constants ensure scale-invariant learning across layers and datasets. Dividing the KL divergence by the **z** dimensionality $R_{z}^{l}$ prevents layers with more **z** units from disproportionately affecting the free energy. Likewise, dividing the prediction error by the data dimension *R*_*X*_ accounts for variability in sensory data size, which helps avoid overfitting to high-dimensional outputs. This practice aligns with stability improvements shown in variational inference literature, including our prior work (Ohata and Tani, 2020).

In our implementation, the meta-prior **w** initially introduced as a global parameter (Equation 2) is extended to layer-specific versions **w**^*l*^ for flexibility (Equation 9). This allows for layer-wise precision modulation, which mirrors the hierarchical structure of cortical processing. While all **w**^*l*^ can be set equal to a global **w**, we allow them to vary to reflect biologically inspired architectures (e.g., Idei et al., 2024), where layers may exhibit different time constants and confidence levels.

By minimizing Equation 9, the posterior inference is performed during network learning and during the perception task. Figure 1 shows a schematic illustration of the posterior inference process of a two-layer PV-RNN model used in the current simulation with an optimization window of three time steps. At every sensory step, an adaptive variable **A** in the window is optimized through multiple epochs of stochastic gradient descent. In the network learning phase, weights and bias parameters θ and ϕ of the generative and inference models, including an adaptive variable **A** for the approximate posterior *z*^*q*^ are jointly optimized. In the perception task phase, network parameters θ and ϕ are fixed, and free energy is minimized at each time step within a dedicated inference window by optimizing only **A** parameterizing the approximate posterior.

#### 2.2.3 Adaptation of meta-prior

The meta-prior **w** is dynamically adapted based on the average prediction error (**er_*sum*_**) over a fixed length time window in the past. When the error decreases below a predefined threshold (**Thr_*L*_**), **w** transitions to a high value (**w**^*H*^), prioritizing top-down generation, which leads to generating MW. This can be intuitively understood from analogy that continuing easy or predictable tasks tends to initiate MW (Peral-Fuster et al., 2023; Seli et al., 2018). Conversely, when the average prediction error exceeds an upper threshold (**Thr_*H*_**), **w** transitions to a low value (**w**^*L*^), enhancing bottom-up inference. The implementation strategy for autonomous meta-prior switching between FS and MW is described in Algorithm 1. Specifically, the probabilistic shifting between the two modes is given by Equations 10, 11, where *Temp* is the temperature, a tunable parameter that can reflect how stochastic or deterministic the system is (see Section 3.2). This probabilistic switching mechanism is inspired by neurobiological models of policy selection under uncertainty. Similar mechanisms, such as the softmax function, are widely used in computational neuroscience and reinforcement learning to simulate probabilistic neural state transitions and action selection (Friston et al., 2014). While the specific threshold values (**Thr_*L*_**, **Thr_*H*_**) are hyperparameters, they are interpreted functionally as reflectors of a minimal or excessive prediction error context, consistent with meta-awareness-triggering mechanisms proposed in prior models (Sandved-Smith et al., 2021). It is highly speculated that this dynamic adaptation should enable autonomous transitions between FS and MW, as will be validated in the simulation experiments detailed in subsequent sections.

**Algorithm 1:** Autonomous meta-prior switching between focus state (FS) and mind-wandering (MW).

1: Initialize meta-prior **w** (either **w**^*L*^ or **w**^*H*^)
2: if **w** == **w**^*L*^ **then**
3: Compute transition probability from FS to MW:
(10) $$P(FS\to MW)=\text{sigmoid}\left(\frac{-(er_{sum}-Thr_{L})}{Temp}\right)$$
4: Generate random number *r*~𝒢(0, 1)
5: if *r* < 𝒫(*FS*→*MW*) **then**
6: Set meta-prior to **w**←**w**^*H*^
7: end **if**
8: else **if** **w** == **w**^*H*^ **then**
9: Compute transition probability from MW to FS:
(11) $$P(MW\to FS)=\text{sigmoid}\left(\frac{er_{sum}-Thr_{H}}{Temp}\right)$$
10: Generate random number *r*~𝒢(0, 1)
11: if *r* < 𝒫(*MW*→*FS*) **then**
12: Set meta-prior to **w**←**w**^*L*^
13: end **if**
14: end **if**

## 3 Experiments and results

### 3.1 Model training

**Figure 2:**
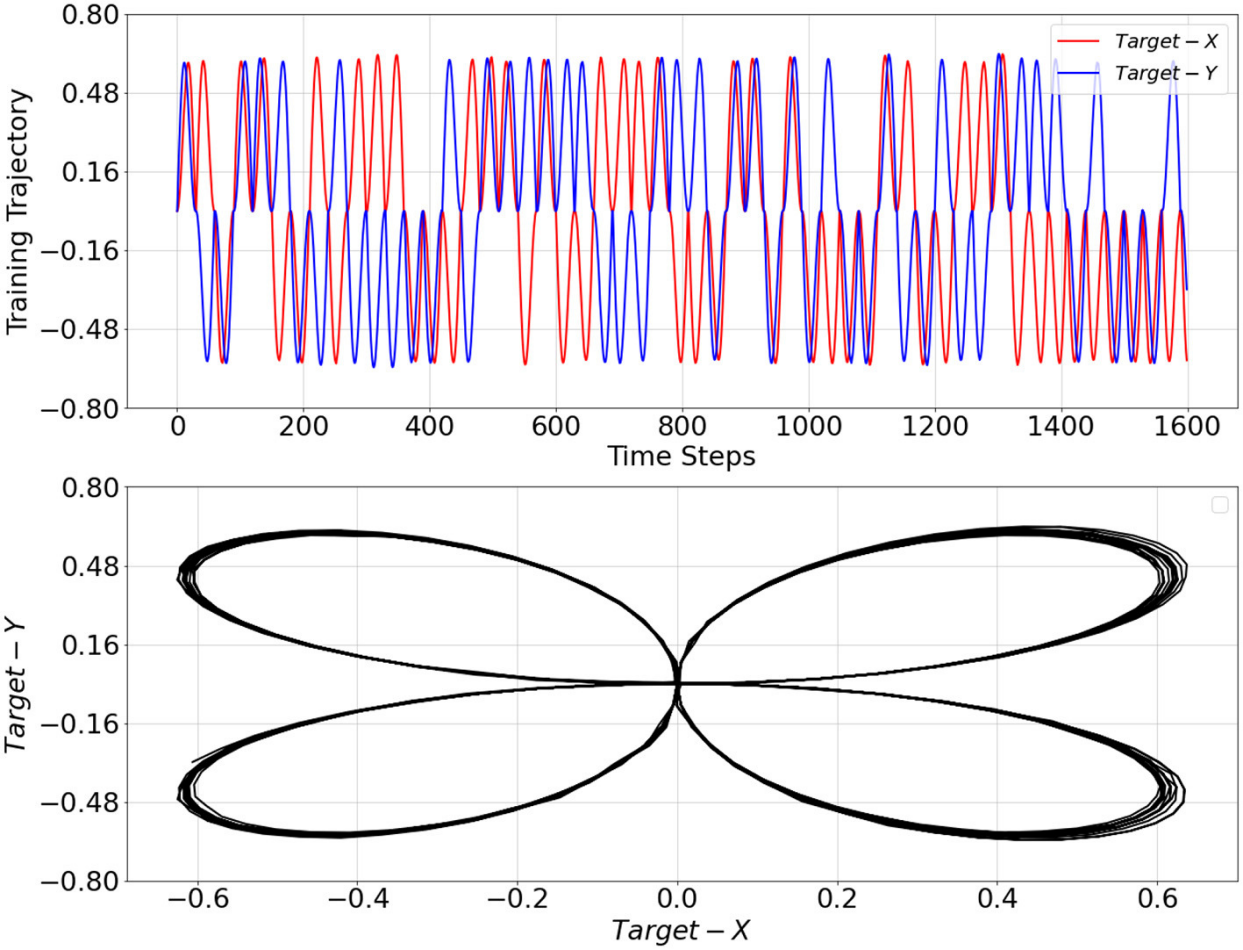
Training trajectory over 1,600 time steps **(top plot)** and its representation in *X*−*Y* space **(bottom plot)**. *Target*−*X* and *Target*−*Y* correspond to the first and second dimensions of the training trajectory, respectively.

First, we trained a PV-RNN with 2-dimensional sensory sequence data. The training data comprised 200 sequences, each containing 3,000 time steps. For preparing those trajectories, we designed 4 different 2-dimensional cyclic patterns, each with a periodicity of 30 time steps. Each trajectory was made of probabilistic switching among these 4 cyclic patterns wherein after one cycle of a particular pattern the same pattern repeats with a probability of 27.27% and the pattern transits to any other pattern with a probability of 72.73% equally. Noise has been added to individual points at randomly spaced intervals. The intervals between noise points are determined by drawing from a normal distribution (mean of 1, standard deviation of 10), providing a variable time step size. At each noise interval, Gaussian noise (mean of 0, standard deviation of 0.003) is added to the current data point, slightly perturbing its coordinates to simulate natural fluctuations without disrupting the cyclic structure. A part of the training trajectory is shown in Figure 2.

The network parameters used for training PV-RNN are listed in Table 1. #**d**, #**z**, τ, **w**^*tr*^ indicates the number of **d** neurons, number of **z** neurons, time constant, and meta-prior during the training phase, respectively.

**Table 1:** PV-RNN training parameters.

**Parameters**	**#d**	**#z**	**τ**	**w^*tr*^**
Layer 1	60	6	3	0.001
Layer 2	30	3	5	0.001

The PV-RNN was trained over 150, 000 epochs minimizing free energy in Equation 9 using the Adam optimizer (Kingma and Ba, 2014) and back-propagation through time (BPTT) (Rumelhart et al., 1985) with learning rate 0.001 to optimize all network parameters of θ and ϕ of the generative and inference model, and the adaptive variable **A** corresponding to each training trajectory.

**Figure 3:**
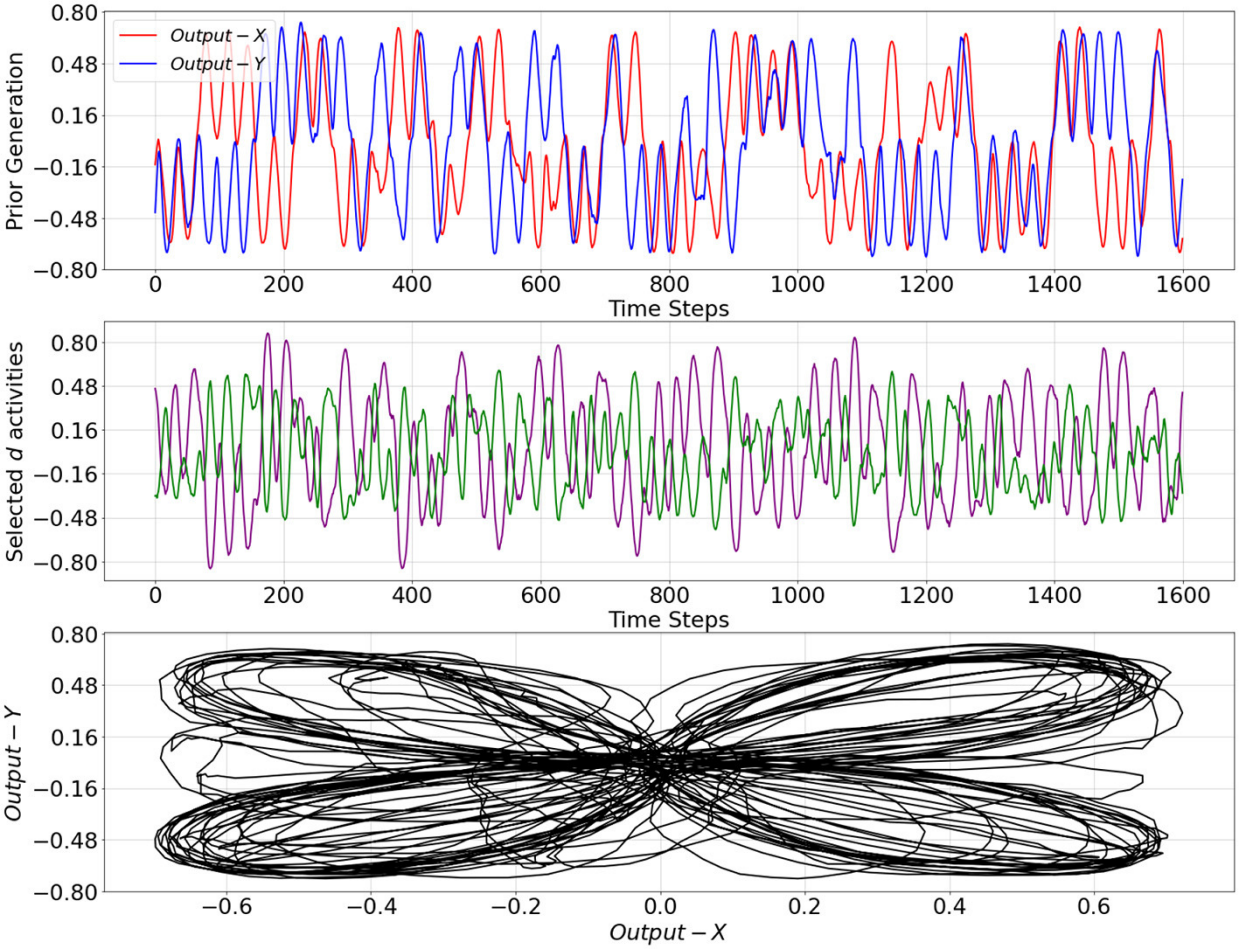
Prior generation over 1,600 time steps under trained model with meta-prior **w**^*tr*^ from Table 1 **(top plot)**, selected activities of the **d** neurons in the bottom layer of the PV-RNN **(middle plot)**, and a representation in *X*−*Y* space **(bottom plot)**. *Output*−*X* and *Output*−*Y* correspond to the first and second dimensions of the prior generation output trajectory, respectively.

The trained network was evaluated on the basis of how well probabilistic transitions in the training data were reflected in the PV-RNN generative process, the so-called prior generation of the PV-RNN, which is conducted without performing the inference of the latent variables with sensory observation. In prior generation, the prior distribution ${\text{z}}_{1}^{p}$ was initialized with a unit Gaussian (Equation 4) and then latent states were recursively computed to generate network output sequences. Figure 3 shows an example of the prior generation outputs over 1,600 time steps. We can see that the patterns shift from one to another, where the two patterns used for training appear randomly. In addition, using a categorizer to discriminate between the two patterns, different prior generation outputs over 10,000 time steps have shown a probability of 28.92% of switching to a different pattern and a probability of 71.08% of staying in the same pattern, which are close to the training dataset.

### 3.2 Testing of perception task

The trained PV-RNN was tested by performing the perception task. In the test, the inference process was performed within the inference window, while one of the trained patterns was used as the target sensory sequence for the inference of the latent variables. The length of the inference window was set to 400 time steps. The adaptation of meta-prior, **w**, during inference with the monitoring of the average reconstruction error over 300 time steps^1^ was carried out using the parameters listed in Table 2.

**Table 2:** PV-RNN testing parameters.

**Parameters**	**w^*L*^**	**w^*H*^**	**Temp**	**Thr_*L*_**	**Thr_*H*_**
Layer 1	0.01	100	0.01	0.15	0.40
Layer 2	0.01	100			

**Figure 4:**
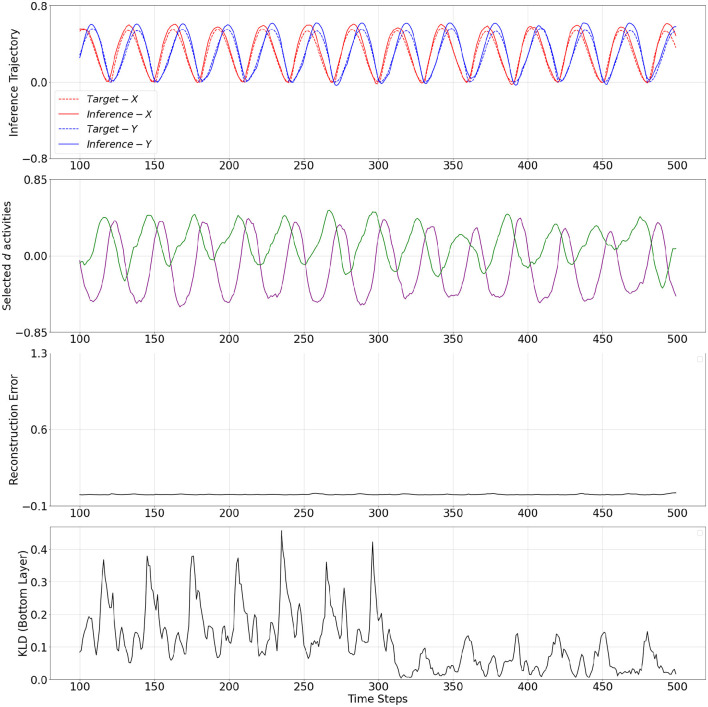
From top to bottom: inference output trajectory with meta-prior **w**^*L*^ from Table 2, selected activities of the **d** neurons in the bottom layer of the PV-RNN, average reconstructions error over the inference window at time step 100, and KL divergence at the PV-RNN bottom layer. *Inference*−*X* and *Inference*−*Y* correspond to the first and second dimensions of the inference output trajectory, respectively.

**Figure 5:**
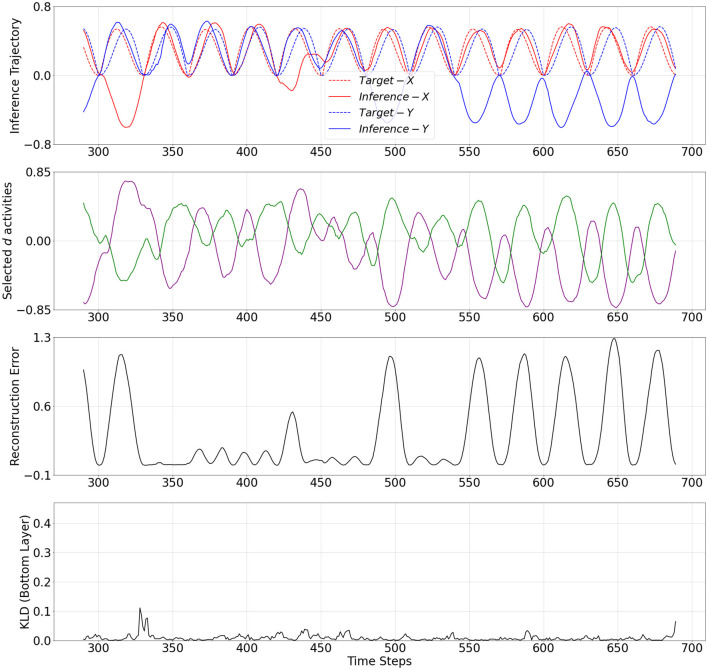
From **top to bottom**: inference output trajectory with meta-prior **w**^*H*^ from Table 2, selected activities of the **d** neurons in the bottom layer of the PV-RNN, average reconstructions error over the inference window at time step 290, and KL divergence at the PV-RNN bottom layer. *Inference*−*X* and *Inference*−*Y* correspond to the first and second dimensions of the inference output trajectory, respectively.

The mechanistic behavior when **w** adapted to low and high values are shown in Figures 4, 5, respectively. The plots show the output trajectory, the target sensory sequence, the average reconstruction error, and KL divergence at the PV-RNN bottom layer for each case. Importantly, no external triggers are used to impose state transitions. Instead, the model is trained on probabilistically switching sequences, allowing it to internalize the statistical structure. During inference, the switching behavior is driven solely by the internal dynamics of the average reconstruction error. Here, the autonomous modulation of meta-prior based on learned internal signals ensures that transitions reflect endogenous state changes, not artificially induced behavior.

In fact, it can be seen in Figure 4 that when **w** adapted to **w**^*L*^, a pattern used for the target sensory sequence is generated well during inference while the average reconstruction error remains low (below 0.05) over the entire inference window. This indicates that adaptation of **w** to **w**^*L*^ enabled the output to accurately reconstruct the target sensory sequence. This period is analogous to a situation of FS.

On the other hand, Figure 5 demonstrates a period when **w** adapted to **w**^*H*^. In this period, the inference trajectory is generated similarly to the prior generation shown in Figure 3. In particular, we can observe in Figure 5 that after a few cycles of one pattern, the inference trajectory generates one of the other patterns in the inference window. Specifically, when **w** changed to **w**^*H*^, KL divergence between prior and inference is more heavily weighted in Equation 9 and, thus, it becomes smaller compared to the KL divergence observed in Figure 4. At the same time, to minimize the free energy, this increase in the meta-prior value allows the prediction error signal to become larger compared to the prediction error computed in Figure 4. As a result, the average reconstruction error increases once the inference trajectory starts to deviate from the target sensory sequence. This observation is analogous to a situation of MW.

Selected **d** activities of the PV-RNN bottom layer during prior generation after training, as well as during inference with adaptation of **w** to low and high values, are shown in Figures 3–5. In both cases of the inference, the correspondence between **d** activity patterns and the output patterns is analogous to that observed during prior generation after training. Specifically, the **d** activities follow a single pattern when **w** adapted to the low value, while the **d** activities alternate between different patterns, closely reflecting the dynamics of the **d** activities seen in the prior generation when **w** adapted to the high value.

**Figure 6:**
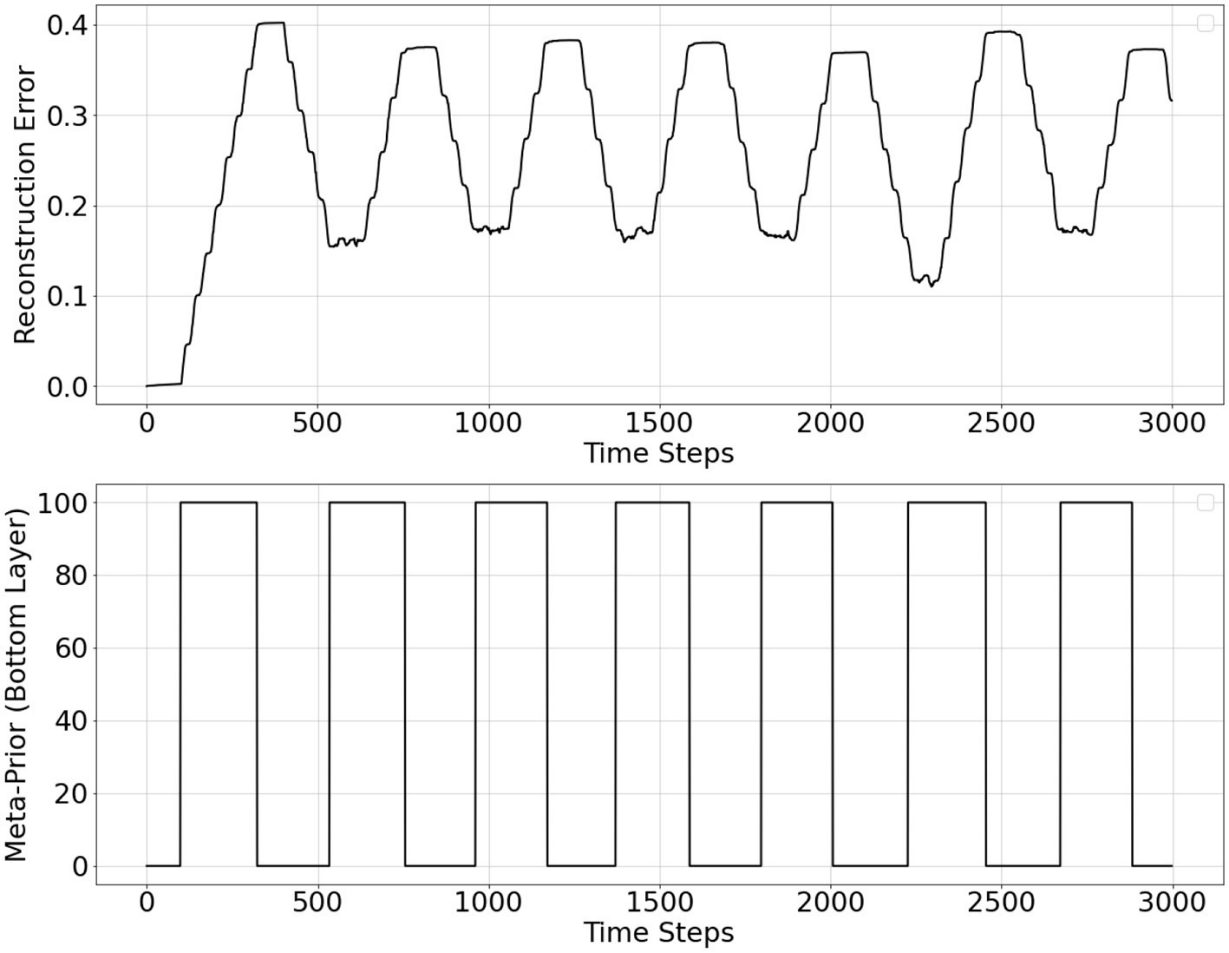
Reconstruction error over inference window computed at each time step **(top plot)** and adaptive meta-prior value (**w**) of the PV-RNN bottom layer over time **(bottom plot)**.

Figure 6 shows the overall behavior of autonomous shifts between two distinct states obtained in the experiments. The plots show the average reconstruction error during inference and meta-prior values of the PV-RNN bottom layer over time. It can be observed that when PV-RNN is under **w**^*H*^, the average reconstruction error increases as close to the high threshold value, which makes the probability of switching from **w**^*H*^ to **w**^*L*^ larger according to Equation 11. Then, **w** is switched to the low value (**w**^*L*^). After this shift, the average reconstruction error continues to decline until it becomes close to the low threshold value, which increases the probability of switching from **w**^*L*^ to **w**^*H*^. **w** is then switched back to the high value. The former case corresponds to the shift from MW to FS and the latter case corresponds to the shift from FS to MW.

**Figure 7:**
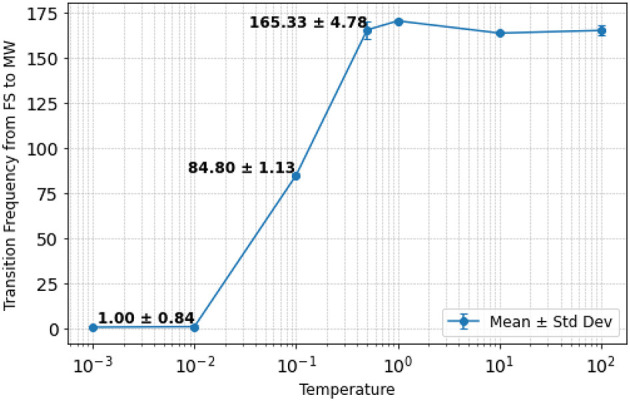
Transition frequency from FS to MW per 1,000 time steps under different temperature values. The mean and standard deviation are displayed for three intermediate cases when temperature is 0.01, 0.10, and 0.50.

Finally, we investigated the effect of changing temperature values on the characteristics of the shifts between FS and MW. For this purpose, we counted the number of transitions that occurred from FS to MW during 1000 steps in the perception test. The results are shown in Figure 7. It can be seen that the transition frequency from FS to MW increases as the temperature increases. In particular, for larger temperature values, the transitions from FS to MW become more frequent (i.e., the system becomes more random) since the probability of switching from FS to MW becomes closer to 50% due to the argument inside the sigmoid function being closer to zero in Equation 10. In contrast, when the temperature is smaller, the transitions from FS to MW become less frequent (i.e., the system becomes more deterministic), which primarily happens when the average reconstruction error reaches the low threshold. For the case study in Figure 6, 0.01 was chosen to be the temperature with a mean of 1.78 transitions from FS to MW per 1,000 time steps.

## 4 Discussion

This study explored the neural mechanisms underlying autonomous shifts between the focused state and mind-wandering through simulation experiments using a newly proposed model based on the free energy principle. The proposed model, an extension of PV-RNN, introduces an online adaptation mechanism for a meta-level parameter, the meta-prior **w**, which is modulated based on the average reconstruction error over a fixed-size past window. Specifically, **w** probabilistically switches to a high value when the average reconstruction error decreases close to a minimal threshold and to a low value when the average reconstruction error increases near a maximal threshold.

In the simulation experiments, the PV-RNN was first trained to generate probabilistic transitions between four distinct cyclic patterns. In the perception task phase, latent variables within the inference window were inferred to minimize the reconstruction error for a given target sensory sequence while adapting **w**. Here, one of the trained cyclic patterns was used as the target sequence.

When **w** shifted to a low value, stronger bottom-up sensory perception dominated, regenerating the observed sensory sequence in the outputs with minimal reconstruction error while allowing larger Kullback-Leibler divergence between the prior and the approximate posterior. This process leads to a state closely aligned with focused attention. In contrast, when **w** shifted to a high value, the approximate posterior is attracted toward the prior by a stronger mean of minimizing the Kullback-Leibler divergence between the prior and the approximate posterior. This allowed stronger top-down processing with less emphasis on sensation, generating relatively large reconstruction error in the inference window. This results in a state resembling mind-wandering.

One may argue that a limitation of the current study is that the proposed model may not account for the phenomenon of becoming consciously aware of MW, which enables redirection of attention back to FS. Previous work in our group (Tani and White, 2022; Tani, 1998) hypothesized that self-consciousness arises from the interplay of top-down predictions and bottom-up sensory inputs, specifically during moments of incoherence between internal predictions and external sensory feedback. In particular, when the system operates smoothly with minimal prediction error, the processes of action and perception may remain unconscious and seamlessly synchronized. However, a large prediction error may disrupt this synchrony, forcing the system to adjust its internal states and expectations to re-establish the system's coherence. This moment of error-driven model recalibration may be accompanied by the emergence of conscious awareness, as the system actively attempts to reconcile conflicting information and restore its predictive model. In the current study, we could consider that self-awareness may be triggered by a prediction error signal accumulated during MW. We, however, speculate that such a hypothetical idea could be improved further by incorporating possible meta-cognitive mechanisms in consciousness.

In this regard, Sandved-Smith et al. (2021) proposes an inference of meta-states from higher levels to lower levels in terms of attention. Particularly, this framework integrates a three-level generative model to simulate how meta-awareness emerges and modulates attention. At the first level, the model represents sensory or perceptual states, encoding external *stimuli*. The second level captures attentional states, such as “focused” or “distracted,” which condition the precision of sensory observations at the first level. The third level introduces meta-awareness states, which monitor and regulate attentional states by dynamically adjusting their precision and transitions. In Sandved-Smith et al. (2021), although the meta-awareness state is shifted manually by manipulating a meta-parameter, by formalizing the hierarchical relationship between meta-awareness and attention, meta-awareness states can modulate confidence in attentional states, which, in turn, influence the precision of sensory observations. This structure allows for inference and control of attentional processes, enabling the agent to recognize transitions in attention, detect MW, and refocus.

Drawing from Sandved-Smith et al. (2021), we describe in the following how the hypothesis proposed by Tani and White (2022) and Tani (1998) based on prediction error could be extended by including the process of inference of a meta-state associated with self-awareness during MW. In particular, a generative model may be included on top of the highest layer of the proposed PV-RNN architecture that receives the average reconstruction error (**er_*sum*_**) as the target signal from the output layer of PV-RNN. This meta-level generative model predicts the average reconstruction error over a time window to minimize free energy. In this internal closed-loop computation, the probabilistic shift between FS and MW is now controlled by the predicted **er_*sum*_** of the meta-level generative model using Equations 10, 11. A meta-level state may be included in this PV-RNN model that is inferred when the mismatch between the predicted **er_*sum*_** and the actual **er_*sum*_** exceeds a certain meta-level threshold at which self-awareness of MW occurs.

In the current proposed work, due to the probabilistic nature of the threshold mechanism for the average reconstruction error, the effective threshold for switching from MW to FS varies dynamically between a lower and higher range. Here, we speculate that consciousness arises specifically when this switching occurs at an effective threshold exceeding a certain “consciousness threshold” (which may be different from the threshold **Thr_*H*_** defined for Equation 11). This perspective explains both the two-stage model (Voss et al., 2018) or the multiple sub-event model (Zukosky and Wang, 2021). In the two-stage model, MW persists until a single transition back to FS occurs at a relatively low “consciousness threshold.” In contrast, the multiple sub-event model allows for repeated, unconscious FS-MW shifts before reaching a higher “consciousness threshold” that triggers conscious awareness. By incorporating this into our proposed PV-RNN framework, we provide a meta-cognitive perspective on how conscious awareness of MW emerges probabilistically, linking predictive coding with varying thresholds of self-awareness based on average reconstruction error.

To enhance the empirical relevance of our model, we now provide a qualitative comparison between its behavioral signatures and well-established findings from experimental studies of mind-wandering (MW). Although our current simulations do not directly incorporate or validate against human neurophysiological data, the model successfully captures several key behavioral characteristics observed in MW literature. These alignments support the potential of our predictive-coding-inspired framework to serve as a mechanistic model of spontaneous attentional dynamics. One robust behavioral marker of MW is increased response time variability during cognitive tasks, as demonstrated by both Smallwood et al. (2008) and Henŕıquez et al. (2016). In our simulations, this phenomenon emerges naturally during periods when the meta-prior shifts to a high value. In this state, the model prioritizes top-down processing, and its output trajectory becomes less constrained by incoming sensory information. This internal mode leads to divergence from the task-aligned trajectory, which would manifest as variability in response behavior if implemented in a real-time embodied system. Thus, the dynamic increase in prediction error and loss of sensory fidelity in the MW state qualitatively parallels the behavioral variability observed in human studies.

The model also accounts for spontaneous alternation between task-focused and mind-wandering states, which has been described as a core feature of the human attentional stream (Zukosky and Wang, 2021; Smallwood and Schooler, 2015). In our framework, transitions between low **w** (FS) and high **w** (MW) modes arise autonomously through probabilistic switching driven by fluctuations in the average reconstruction error. Crucially, these shifts are not externally triggered but emerge from the internal dynamics of prediction error accumulation, reflecting a self-organizing process akin to that observed in subjective reports of spontaneous MW episodes.

In addition, our model can reproduce temporal dynamics associated with MW frequency over extended task performance. Empirical studies such as Zanesco et al. (2024) have shown that MW tends to increase with time-on-task, often attributed to reduced cognitive engagement or habituation. In our simulations, prolonged accurate predictions lead to a steady decrease in the average reconstruction error, which in turn increases the probability of transitioning into the MW state. This behavior provides a computational account for the time-dependent drift toward MW observed in attentional tasks, as the internal model becomes overconfident and sensory information is deprioritized.

Furthermore, the model can reflect the empirically observed nonlinear dependence of MW on task difficulty (Robison et al., 2020; Xu and Metcalfe, 2016). When the task is too easy, prediction error remains low and the model is prone to transition into the high **w** mode, corresponding to disengagement or MW. Under moderately challenging conditions, the model sustains engagement as prediction error fluctuates within a manageable range. When the task becomes highly difficult, persistent prediction error may trigger frequent or prolonged re-entries into MW-like states, reflecting cognitive overload or motivational disengagement. These qualitative dynamics are consistent with behavioral studies showing that MW is minimized at intermediate levels of task challenge and increases under both underload and overload conditions.

Notably, Shinagawa and Yamada (2025) recently proposed a homeostatic reinforcement learning (HRL) framework to model mind-wandering under structured task conditions, particularly the Sustained Attention to Response Task (SART). Although our model differs architecturally (i.e., being grounded in predictive coding and variational inference), it shares the key objective of capturing autonomous cognitive state transitions without external supervision. Both models simulate internal modulation mechanisms (e.g., prediction error and homeostatic drives) that regulate attentional shifts between task-focused and internally guided states.

Although we have not explicitly implemented a SART-like structure in our simulation setup, our model's probabilistic state switching and its sensitivity to internal error dynamics mirror the mechanisms used in HRL-SART simulations. We believe that adapting the extended PV-RNN to directly simulate or align with task-based paradigms like SART represents a valuable direction for future work. Such an extension would enable quantitative comparison with behavioral and physiological data, helping bridge synthetic modeling with empirical paradigms in attention and mind-wandering research.

Taken together, these qualitative comparisons suggest that our extended PV-RNN model captures essential properties of MW dynamics reported in behavioral studies. The internal modulation of the meta-prior based on reconstruction error not only supports autonomous shifts between attentional states but also aligns with empirical observations in timing, variability, and task-context sensitivity of mind-wandering.

As for neurophysiological validation, we acknowledge that such comparison remains an open and important direction for future work. The current study is intended as a principle-level computational investigation that explores the feasibility of autonomous attentional modulation via predictive coding mechanisms. Nevertheless, future extensions of the model could examine whether its internal state transitions, particularly changes in the meta-prior, correspond to physiological markers of MW such as fluctuations in EEG alpha-band activity, pupil diameter, or large-scale fMRI network reconfigurations involving the default mode and salience networks. Such investigations would provide stronger links between the model's theoretical mechanisms and observed neural phenomena.

Moreover, numerous studies have indicated that MW during the resting state is intricately linked to the functional organization and dynamics of brain networks, particularly the default network (DN), central executive network (CEN), and salience network (SN) (Mason et al., 2007; Godwin et al., 2017; Denkova et al., 2019). However, the current study does not model interactions between such distinct networks. Reservoir computing may also be utilized as a component of the brain network, which has been proposed to be a neural basis in the cortex (Yonemura and Katori, 2024). Extending the model to incorporate dynamic interactions among these networks would provide a tighter connection to established neuroscientific findings on resting-state phenomena and offer deeper insights into mind-wandering.

Finally, beyond theoretical modeling, our approach may hold potential for practical applications. For instance, adaptive learning systems could use internal signals such as prediction error or meta-prior shifts to detect mind-wandering episodes and adjust content delivery in real time. Similarly, our model could be a mental health or mindfulness support tool to inform computational accounts of attentional fluctuations, and guide human-AI systems toward more flexible attentional control. We view these applications as promising but preliminary, and future work will be needed to integrate the model into real-world tasks and evaluate its practical effectiveness.

## Data Availability

The proposed PV-RNN and dataset generated for this study can be found in the following GitHub repository: https://github.com/oist-cnru/tiny/tree/mw_develop. Further inquiries can be directed to the corresponding author.

## Footnotes

^1^For the implementation strategy described in Algorithm 1, the length of the inference window and time window for computing the average reconstruction error can be considered design decisions and do not need to be the same length. This choice may depend on how the high and low thresholds are defined, which impact the probability of transition from FS to MW (and vice-versa) and, thus, the expected probabilistic behavior of the system. For instance, an average reconstruction error computed over a small time window may not reach or may be too far from a desired threshold. In this scenario, the probability of staying in the current state (FS or MW) would remain large over the entire simulation time.
